# Evaluation of Molecular Epidemiology, Clinical Characteristics, Antifungal Susceptibility Profiles, and Molecular Mechanisms of Antifungal Resistance of Iranian *Candida parapsilosis* Species Complex Blood Isolates

**DOI:** 10.3389/fcimb.2020.00206

**Published:** 2020-05-21

**Authors:** Amir Arastehfar, Farnaz Daneshnia, Mohammad Javad Najafzadeh, Ferry Hagen, Shahram Mahmoudi, Mohammadreza Salehi, Hossein Zarrinfar, Zahra Namvar, Zahra Zareshahrabadi, Sadegh Khodavaisy, Kamiar Zomorodian, Weihua Pan, Bart Theelen, Markus Kostrzewa, Teun Boekhout, Cornelia Lass-Flörl

**Affiliations:** ^1^Yeast Biodiversity Department, Westerdijk Fungal Biodiversity Institute, Utrecht, Netherlands; ^2^Department of Parasitology and Mycology, School of Medicine, Mashhad University of Medical Sciences, Mashhad, Iran; ^3^Department of Medical Microbiology, University Medical Center Utrecht, Utrecht, Netherlands; ^4^Laboratory of Medical Mycology, Jining No. 1 People's Hospital, Jining, China; ^5^Department of Medical Parasitology and Mycology, School of Medicine, Iran University of Medical Sciences, Tehran, Iran; ^6^Department of Infectious Diseases and Tropical Medicine, Faculty of Medicine, Tehran University of Medical Sciences, Tehran, Iran; ^7^Allergy Research Center, Mashhad University of Medical Sciences, Mashhad, Iran; ^8^Department of Microbiology, School of Biological Sciences, Islamic Azad University, Tehran, Iran; ^9^Basic Sciences in Infectious Diseases Research Center, Shiraz University of Medical Sciences, Shiraz, Iran; ^10^Department of Medical Parasitology and Mycology, School of Public Health, Tehran University of Medical Sciences, Tehran, Iran; ^11^Medical Mycology, Shanghai Changzheng Hospital, Second Military Medical University, Shanghai, China; ^12^Bruker Daltonik GmbH, Bremen, Germany; ^13^Institute of Biodiversity and Ecosystem Dynamics, University of Amsterdam, Amsterdam, Netherlands; ^14^Division of Hygiene and Medical Microbiology, Medical University of Innsbruck, Innsbruck, Austria

**Keywords:** Iran, candidemia, *Candida parapsilosis*, AFLP genotyping, high mortality rate, *Candida orthopsilosis*

## Abstract

Clonal expansion of fluconazole resistant (FLZ-R) *Candida parapsilosis* isolates is increasingly being identified in many countries, while there is no study exploring the antifungal susceptibility pattern, genetic diversity, and clinical information for Iranian *C. parapsilosis* blood isolates. *Candida parapsilosis* species complex blood isolates (*n* = 98) were recovered from nine hospitals located in three major cities, identified by MALDI-TOF MS, and their genetic relatedness was examined by AFLP fingerprinting. Antifungal susceptibility testing followed CLSI-M27-A3 and *ERG11, MRR1* and hotspots 1/2 (HS1/2) of *FKS1* were sequenced to assess the azole and echinocandin resistance mechanisms, respectively. Ninety-four *C. parapsilosis* and four *Candida orthopsilosis* isolates were identified from 90 patients. Only 43 patients received systemic antifungal drugs with fluconazole as the main antifungal used. The overall mortality rate was 46.6% (42/90) and death mostly occurred for those receiving systemic antifungals (25/43) relative to those not treated (17/47). Although, antifungal-resistance was rare, one isolate was multidrug-resistant (FLZ = 16 μg/ml and micafungin = 8 μg/ml) and the infected patient showed therapeutic failure to FLZ prophylaxis. Mutations causing azole and echinocandin resistance were not found in the genes studied. AFLP revealed five genotypes (G) and G1 was the main one (59/94; 62.7%). Clinical outcome was significantly associated with city (*P* = 0.02, α <0.05) and Mashhad was significantly associated with mortality (*P* = 0.03, α <0.05). Overall, we found a low level of antifungal resistance for Iranian *C. parapsilosis* blood isolates, but the noted MDR strain can potentially become the source of future infections and challenge the antifungal therapy in antifungal-naïve patients. AFLP typing results warrants confirmation using other resolutive typing methods.

## Introduction

The changing epidemiological landscape of candidemia driven by overuse of prophylactic antifungal drugs has resulted in an increasing incidence of non-*albicans Candida* (NAC) species (Sanglard, 2019). Presently, microbiologists and clinicians are heavily focused on multi-drug resistant *Candida parapsilosis* and *Candida auris* (Colombo et al., 2017), but an increasing number of publications are casting light on the importance of other NAC species, as well (Chakrabarti et al., 2015; Singh et al., 2019). Among these NAC species, *Candida parapsilosis* is the first to third common cause of candidemia depending on age, geographical location, and patient category (Chan et al., 2015; Da Matta et al., 2017; Sun et al., 2019). The biofilm production (Marcos-Zambrano et al., 2014; Larkin et al., 2018), which is viable for weeks on plastic surfaces (Thomaz et al., 2018), and resilience of *C. parapsilosis* to stay in clinical settings is reminiscent of that of *C. auris* (Choi et al., 2018). Apart from being less susceptible to echinocandins (Garcia-Effron et al., 2008), fluconazole resistant *C. parapsilosis* isolates have emerged in India (Singh et al., 2019), South Korea (Choi et al., 2018), Kuwait (Asadzadeh et al., 2017a), USA (Berkow et al., 2015; Grossman et al., 2015), and Brazil (Souza et al., 2015; Thomaz et al., 2018). Additionally, the closely related cryptic species of *C. parapsilosis*, i.e., *C. orthopsilosis*, is linked to numerous clinical failures (Wessel et al., 2013; Oliveira et al., 2014; Heslop et al., 2015; Charsizadeh et al., 2018b), and implicated in a wide range of clinical manifestations, including superficial infections (Feng et al., 2012), septic arthritis (Heslop et al., 2015), keratitis (Wessel et al., 2013), and fatal invasive bloodstream infections (Choi et al., 2010).

Apart from overexpression of efflux pumps such as Cp*CDR1* and Cp*MDR1* (Grossman et al., 2015; Souza et al., 2015), in the majority of cases specific genetic alterations in Cp*ERG11* (Grossman et al., 2015; Souza et al., 2015; Choi et al., 2018; Thomaz et al., 2018; Singh et al., 2019) and in some cases in Cp*MRR1* can result in azole resistance in *C. parapsilosis* (Grossman et al., 2015; Choi et al., 2018). Moreover, a naturally occurring amino acid substitution in HS1 of *FKS1* proved to be accountable for high MIC values of echinocandin in this species complex (Garcia-Effron et al., 2008).

*Candida parapsilosis* is easily spread through the hands of healthcare workers (HCWs) and some studies revealed that specific azole resistant genotypes of this species are able to stay in a dormancy phase for a long period of time and can be the source of future clonal outbreaks and cause azole-recalcitrant infections in patients that have not been exposed previously to this drug (Choi et al., 2018; Singh et al., 2019). As a result, genotypic analysis could be an important guide to control infections caused by this species. Among genotypic techniques, AFLP fingerprinting has been associated with a higher resolution than the laborious and expensive multi-locus sequence typing (MLST) technique (Asadzadeh et al., 2017b). Moreover, studies showed satisfactory resolution of AFLP to evaluate the genetic relatedness of *C. auris* (Prakash et al., 2016) and *C. parapsilosis* species complex isolates (Tavanti et al., 2010). Ease of optimization and universality of the primers and experimental conditions are the advantages of this technique (Restrepo et al., 2018).

Lack of knowledge about clinical outcome, genotypic diversity, antifungal susceptibility profiles, and the corresponding molecular mechanism of antifungal resistance at a national scale for *C. parapsilosis* bloodstream isolates, prompted us to conduct a multicenter study to fill those gaps in Iran. Clarifying those factors will aid in a better clinical management and provide insights about the extent of the necessity of implementation of infection control strategies.

## Methods

### Ethical Approval

Candidemia studies, from which *Candida parapsilosis* isolates were obtained, conducted in Tehran, Shiraz, and Mashhad were granted with ethical approval (IR.SUMS.REC.1397.365, IR.MUMS.fm.REC.1397.268, IR. TUMS.SPH.REC.1396.4195). Isolates of *C. parapsilosis* were assigned with numerical codes to anonymize the patient's identity.

### Definitions, Study Design, and Growth Conditions

Definition of candidemia was in accordance with the revised definition of European Organization for Research and Treatment of Cancer (De Pauw et al., 2008). Isolates recovered within <30 days and those recovered within >30 days were considered as repetitive isolates and new cases, respectively (Blyth et al., 2009). The entire stay of respective patients following admission to discharge was considered for hospitalization duration, but not after the isolation of *C. parapsilosis* from blood samples (as this data was not available for all patients included in this study). *Candida parapsilosis* bloodstream isolates were retrospectively recovered from nine hospitals (2015–2019) located in three main metropolitan cities, including Tehran (two hospitals), Shiraz (two hospitals), and Mashhad (five hospitals). The majority of the isolates were from Mashhad (*n* = 60, 61.2%), followed by Tehran (*n* = 24, 24.5%), and Shiraz (*n* = 14, 14.3%). All patients manifesting candidemia due to *C. parapsilosis* species complex without any restriction were included in our study. Blood samples were incubated in Bactec devices (Becton Dickinson, MD, USA) and recovered isolates were grown on Sabouraud dextrose agar at 37°C for 24–48 h, and to identify samples with mixed *Candida*/yeast species, they were subcultured onto chromogenic agar (Candiselect, Bio-Rad, Hercules, CA, USA) at 37°C for 48 h.

### Identification

Isolates were identified by MALDI-TOF MS using the full-extraction method [29] and those identified as *C. orthopsilosis* or *C. metapsilosis* were further confirmed by Sanger sequencing using ITS5 and LR5 primers targeting part of the 28S and internal transcribed spacer (ITS) rDNA (Stielow et al., 2015). The DNA samples were extracted using a previously described CTAB-phenol/chloroform protocol (Theelen et al., 2001).

### Primer Design and Sequencing of Cp*ERG11, CpMRR1*, and HS1 and HS2 of Cp*FKS1*

PCR was performed in a final volume of 50 μl. All primers used in this study, the PCR programs, and the PCR ingredients are listed in Supplementary Table 1.

Sanger sequencing was performed for the aforementioned genes, contigs were assembled by SeqMan Pro (DNASTAR, Madison, USA), and the obtained sequences along with references were aligned with MEGA v7.0 (Temple University, Philadelphia, USA) (Kumar et al., 2016). Sequences of *ERG11* and *MRR1* were compared with the corresponding reference wild-type *ERG11* sequences of ATCC 22019 (GQ302972) and CDC317 wild-type *MRR1* sequence (HE605205), respectively (Berkow et al., 2015). As for the sequences of *FKS1* HS1 and HS2 they were compared with those previously reported (Garcia-Effron et al., 2008). Although, Y132F is mainly found in FLZ-R isolates, a recent study identified this mutation in an isolate showing FLZ-intermediate phenotype (Singh et al., 2019). Moreover, according to our experience with *C. glbarata*, not all echinocandin susceptible isolates are wild-type in HS and they can harbor well-known accountable mutations in their HS regions (Arastehfar et al., 2020b). Therefore, *ERG11* and HS1 and HS2 of *FKS1* were sequenced for all isolates included in this study, while *MRR1* was sequenced for only selective number of isolates (see Results).

### Evaluation of Genotypic Diversity Using AFLP

In order to check the genotypic diversity, a previously described AFLP fingerprinting method was used (Arastehfar et al., 2019a). Diluted PCR products were analyzed by capillary electrophoresis on an ABI 3730xL Genetic Analyzer (ThermoFisher Scientific, Waltham, MA, USA) and the obtained data were analyzed by Bionumerics software v7.6.2 (Applied Math, Sint-Martens-Latem, Belgium). Analysis was based on fragment size and its presence/absence among isolates tested and included standard Pearson and unweighted pair group method with averages (UPGMA) as performed before (Prakash et al., 2016). Reference and type strains of *C. parapsilosis* (CBS 604, CBS 1818, CBS 1954, CBS 2195, and CBS 2917), *C. metapsilosis* (CBS 2315, CBS 2916, and CBS 10907), and *C. orthopsilosis* (CBS 10906) were included for comparative purposes.

### Antifungal Susceptibility Testing

The CLSI broth microdilution (CLSI-BMD) method of M27-A3/S4 was used for antifungal susceptibility testing (AFST) (Clinical and Laboratory Standards Institute, M27-A3, 2008; Clinical and Laboratory Standards Institute, M27-S4, 2012). AFST included the following antifungal drugs, amphotericin B (AMB), fluconazole (FLZ), voriconazole (VRZ), itraconazole (ITZ) all from (Sigma-Aldrich, St. Louis, MO, U.S.A), micafungin (MFG) (Astellas, Munich, Germany) and anidulafungin (ANF) (Pfizer, NY, USA). Reference strains of *C. parapsilosis* (ATCC 22019) and *C. krusei* (ATCC 6258) were used for quality control purposes. Due to interlaboratory variation, caspofungin was not used in this study (Espinel-Ingroff et al., 2013). Plates containing antifungal drugs and isolates were incubated at 37°C for 24 h and data were recorded visually. MIC data were interpreted based on CLSI M60 (Clinical and Laboratory Standards Institute, M60, 2017). Isolates showing a minimum inhibitory concentration (MIC) ≥8 μg/ml were noted as resistant to FLZ, MFG, and ANF, while those with VRZ MIC ≥1 μg/ml were regarded resistant (Clinical and Laboratory Standards Institute, M60, 2017). AMB and ITZ MIC values were interpreted as epidemiological cut-off values (ECV), where non-wild-types (NWT) isolates had a MIC >2 and >0.5 μg/ml, respectively (Clinical and Laboratory Standards Institute, M60, 2017).

### Deposition of Isolates and Sequences

Isolates of *C. orthopsilosis* were deposited in the culture collection of the Westerdijk Fungal Biodiversity Institute (CBS 15892, CBS 15878, CBS 15879, CBS 15862). Additionally, sequences of *ERG11, MRR1* and *FKS1* HS1 and HS2 were submitted to GenBank with the following accession numbers MK513945-MK514041, MT019513-MT019524, MK532043-MK532140, and MK532141-MK532237, respectively.

### Statistical Analysis

All statistical analyses included in this study were performed by SPSS software v24 (SPSS Inc. Chicago, IL, USA) and presented in Supplementary Table 2. The Chi-square test was used to find the association between clinical outcome (death or survival), genotypes, and cities involved. In order to assess the association of hospitalization duration and encountered genotypes the Kruskal-Wallis Test was used. The association of genotypes with death was assessed using logistic regression and path analysis. Patients with more than two isolates belonging to various genotypes were not considered for statistical analysis. Variables showing *P* values <0.05 were considered statistically significant.

## Results

### Clinical Profiles

*Candida parapsilosis* accounted for the vast majority of the blood isolates (*n* = 93; 94.9% from 86 patients), followed by *C. orthopsilosis* (*n* = 3; 3%) and one patient was concurrently infected with both *C. parapsilosis* and *C. orthopsilosis* (98 isolates from 90 patients) (Table 1, Supplementary Table 3). Five patient were infected with multiple *C. parapsilosis* isolates (*n* = 12) (three patients with two isolates including 27-BC, 60-BC, and 111-BC vs. two patients with three isolates, including 79-BC and 97-BC) among which only 79-2BC was isolated >30 days apart and considered as new case and the rest as repetitive isolates. There was no difference for *C. parapsilosis* candidemia between males (*n* = 45; 50%) and females (*n* = 45; 50%) (clinical data were calculated per patient number). The vast majority of patients were admitted to ICU (*n* = 44; 48.9%), followed by general ward (*n* = 24; 26.7%), surgery (*n* = 8; 8.9%), and others (*n* = 14; 15.6%) (Supplementary Table 3). The most prevalent risk factors were broad-spectrum antibiotic usage (*n* = 84; 93.3%), CVC insertion (*n* = 78; 86.7%), mechanical ventilation (*n* = 35; 38.9%), surgery (*n* = 30; 33.3%) among which 15.6% were abdominal (*n* = 14), parenteral nutrition (*n* = 25; 27.8%), neutropenia (*n* = 15; 16.7%), and administration of immunosuppressive drugs (*n* = 12; 13.3%) (Supplementary Table 3). Diabetes (*n* = 19; 21.1%), abdominal events (*n* = 19; 21.1%), vascular and heart events and chronic lung diseases (each *n* = 16; 17.8%), leukemia (*n* = 11; 12.2%), and concomitant bacteremia (*n* = 10; 11.1%) were the most encountered underlying conditions (Supplementary Table 3). The median of hospitalization duration was 39 days. Only 47.7% of the patients (*n* = 43) were treated with systemic antifungal drugs, among whom 72.1% (*n* = 31) and 27.9% (*n* = 12) received a single or more than one systemic antifungal drugs (but not in combination) during the course of treatment, respectively. Among those receiving single antifungal treatment (*n* = 31), FLZ was the most widely used antifungal (*n* = 15; 48.4%) followed by AMB (*n* = 9; 29%), CSP (*n* = 6; 19.3%), and VRZ (*n* = 1; 3.2%). The overall mortality rate was 46.6% (*n* = 42). Surprisingly, death occurred for the vast majority of those receiving systemic antifungals (25/43) (17/31 receiving single antifungals and 8/12 received more than one antifungal), while patients not treated with systemic antifungals mostly recovered (30/47; 63.8%). Per antifungal, AMB (6/9; 66.6%) and FLZ (10/15; 66.6%) showed the highest rate of mortality, while those treated with CSP (5/6; 83.3%) mostly recovered (the only patient receiving VRZ survived) (Supplementary Table 3). The highest death count was significantly observed in Mashhad (33/59; 55.9%) (Chi-square, two-tailed, *P* = 0.013), while lower mortality rates occurred in Tehran (7/17; 36.8%) and Shiraz (2/14; 14.3%). At the species level, death occurred in 66.7% of patients infected with *C. orthopsilosis* (2/3) and 45.3% of those infected with *C. parapsilosis* (39/86) (Supplementary Table 3). Death occurred for the only patient simultaneously infected with both *C. parapsilosis* and *C. orthopsilosis*.

**Table 1:** Antifungal susceptibility testing data and sequencing of genes conferring resistance to echinocandins (HS1 and HS2 of *FKS1*) and azoles (*ERG11*).

**Patient #**	**Species**	**Genotype**	**MIC values (μg/ml)**						**ERG11**	**MRR1**	**HS1 FKS1**	**HS2 FKS2**
			**FLZ**	**VRZ**	**ITZ**	**MFG**	**AFG**	**AMB**				
1BC	*C. parapsilosis*	G2	0.25	0.015	0.5	2	2	1	A740R (D247G)	ND	FLTLSLRDA	DWIRRYTL
5BC	*C. parapsilosis*	G3	0.25	0.015	0.03	0.5	1	0.5	A740R (D247G), G1193T (R398I)	ND	FLTLSLRDA	DWIRRYTL
8-1BC	*C. parapsilosis*	G1	0.25	0.015	2	0.5	1	0.5	C168T	ND	FLTLSLRDA	DWIRRYTL
17BC	*C. parapsilosis*	G2	1	0.125	0.25	2	2	0.5	WT	ND	FLTLSLRDA	DWIRRYTL
20BC	*C. parapsilosis*	G3	0.25	0.015	0.06	0.5	1	0.03	A740R (D247G)	ND	FLTLSLRDA	DWIRRYTL
27-1BC	*C. parapsilosis*	G1	0.5	0.06	0.25	2	2	1	WT	ND	FLTLSLRDA	DWIRRYTL
27-2BC	*C. parapsilosis*	G2	0.5	0.06	0.25	2	2	1	WT	ND	FLTLSLRDA	DWIRRYTL
30BC	*C. parapsilosis*	G2	0.5	0.03	0.125	0.5	1	1	WT	ND	FLTLSLRDA	DWIRRYTL
48BC	*C. orthopsilosis*	G2Orth	0.25	0.03	0.06	0.5	1	0.25	WT	ND	FLTLSLRDA	DWVRRYTL
60-1BC	*C. parapsilosis*	G3	2	0.03	0.125	2	2	0.5	WT	A231G	FLTLSLRDA	DWIRRYTL
60-2BC	*C. parapsilosis*	G1	2	0.015	0.125	1	2	0.25	WT	A231G	FLTLSLRDA	DWIRRYTL
64BC	*C. parapsilosis*	G3	0.125	<0.015	0.06	0.5	1	0.5	WT	WT	FLTLSLRDA	DWIRRYTL
67BC	*C. parapsilosis*	G2	1	0.03	0.125	1	2	0.25	WT	ND	FLTLSLRDA	DWIRRYTL
79-1BC	*C. parapsilosis*	G1	0.5	0.015	0.06	2	2	0.25	C168T	ND	FLTLSLRDA	DWIRRYTL
79-2BC	*C. parapsilosis*	G2	0.5	<0.015	0.06	0.5	1	0.5	WT	ND	FLTLSLRDA	DWIRRYTL
79-3BC	*C. parapsilosis*	G4	0.5	<0.015	0.06	0.5	1	1	WT	ND	FLTLSLRDA	DWIRRYTL
97-1BC	*C. parapsilosis*	G1	0.5	0.03	0.125	2	2	0.25	WT	ND	FLTLSLRDA	DWIRRYTL
97-2BC	*C. parapsilosis*	G1	0.25	0.03	0.125	2	2	0.25	WT	ND	FLTLSLRDA	DWIRRYTL
97-3BC	*C. parapsilosis*	G2	0.25	0.03	0.125	2	2	0.5	G747C	ND	FLTLSLRDA	DWIRRYTL
101BC	*C. parapsilosis*	G1	1	0.125	0.25	2	4	1	WT	ND	FLTLSLRDA	DWIRRYTL
106BC	*C. parapsilosis*	G1	0.25	0.03	0.125	1	1	0.25	WT	ND	FLTLSLRDA	DWIRRYTL
111-1BC	*C. parapsilosis*	G2	0.25	<0.015	0.06	0.5	1	1	WT	WT	FLTLSLRDA	DWIRRYTL
111-2BC	*C. parapsilosis*	G3	0.25	<0.015	0.125	0.5	1	1	WT	ND	FLTLSLRDA	DWIRRYTL
131BC	*C. parapsilosis*	G3	16	0.25	0.5	8	4	0.06	WT	WT	FLTLSLRDA	DWIRRYTL
SU92	*C. parapsilosis*	G2	0.5	0.25	0.015	0.5	0.5	1	WT	ND	FLTLSLRDA	DWIRRYTL
SU109	*C. parapsilosis*	G2	0.25	<0.015	0.125	0.5	1	1	WT	ND	FLTLSLRDA	DWIRRYTL
SU159	*C. parapsilosis*	G1	0.5	0.015	0.25	0.5	2	1	A740R (D247G)	ND	FLTLSLRDA	DWIRRYTL
SU225	*C. parapsilosis*	G1	0.5	<0.015	0.03	1	2	0.5	WT	ND	FLTLSLRDA	DWIRRYTL
SU236	*C. orthopsilosis*	NA	0.125	<0.015	0.25	0.5	1	0.25	WT	ND	FLTLSLRDA	DWVRRYTL
SU237	*C. parapsilosis*	G1	0.25	0.015	0.125	0.5	1	1	WT	ND	FLTLSLRDA	DWIRRYTL
SU242	*C. parapsilosis*	G1	0.5	<0.015	0.06	0.5	1	0.5	WT	ND	FLTLSLRDA	DWIRRYTL
SU243	*C. parapsilosis*	G1	0.25	<0.015	0.06	1	1	0.5	WT	WT	FLTLSLRDA	DWIRRYTL
SU251	*C. parapsilosis*	G1	0.25	<0.015	0.03	0.5	1	0.125	WT	ND	FLTLSLRDA	DWIRRYTL
SU255	*C. parapsilosis*	G1	0.25	<0.015	0.125	0.5	1	0.5	WT	ND	FLTLSLRDA	DWIRRYTL
SU259	*C. parapsilosis*	G1	0.5	0.03	0.125	1	1	0.25	WT	ND	FLTLSLRDA	DWIRRYTL
SU266-2	*C. parapsilosis*	G5	0.25	<0.015	0.03	1	2	1	G1193T (R398I)	ND	FLTLSLRDA	DWIRRYTL
SU273	*C. parapsilosis*	G3	0.25	0.015	0.125	0.5	1	1	WT	ND	FLTLSLRDA	DWIRRYTL
SU276	*C. parapsilosis*	G3	0.5	<0.015	0.03	0.5	2	0.5	G266T (G89V)	WT	FLTLSLRDA	DWIRRYTL
N1W	*C. parapsilosis*	G3	0.5	0.06	0.5	1	0.5	0.25	C168T	A3080R^*^	FLTLSLRDA	DWIRRYTL
N1R	*C. orthopsilosis*	G4Orth	0.25	0.03	0.06	0.25	1	0.25	WT	ND	FLTLSLRDA	DWVRRYTL
N16	*C. parapsilosis*	G1	0.25	0.015	0.125	1	0.5	0.06	WT	ND	FLTLSLRDA	DWIRRYTL
N25	*C. parapsilosis*	G1	0.25	<0.015	0.125	1	1	0.25	C168T	ND	FLTLSLRDA	DWIRRYTL
N59	*C. parapsilosis*	G3	0.25	0.015	0.125	0.5	2	0.25	G1193T (R398I)	ND	FLTLSLRDA	DWIRRYTL
N60	*C. parapsilosis*	G1	0.5	<0.015	0.03	0.5	0.5	0.5	WT	ND	FLTLSLRDA	DWIRRYTL
N61	*C. parapsilosis*	G1	1	<0.015	0.25	0.25	0.5	0.25	WT	ND	FLTLSLRDA	DWIRRYTL
N63	*C. parapsilosis*	G1	1	0.015	0.5	0.25	2	0.25	WT	ND	FLTLSLRDA	DWIRRYTL
N65	*C. parapsilosis*	G1	0.25	0.25	0.25	0.5	4	0.5	WT	ND	FLTLSLRDA	DWIRRYTL
N79	*C. parapsilosis*	G1	0.25	<0.015	0.125	0.5	0.5	1	WT	ND	FLTLSLRDA	DWIRRYTL
N80	*C. parapsilosis*	G2	0.25	<0.015	0.25	0.5	1	0.25	WT	ND	FLTLSLRDA	DWIRRYTL
N81	*C. parapsilosis*	G3	0.125	<0.015	0.25	0.5	2	0.25	WT	ND	FLTLSLRDA	DWIRRYTL
N82	*C. parapsilosis*	G1	0.25	<0.015	0.03	0.5	4	0.125	WT	ND	FLTLSLRDA	DWIRRYTL
N83	*C. parapsilosis*	G1	0.5	<0.015	0.03	1	1	0.25	WT	ND	FLTLSLRDA	DWIRRYTL
N84	*C. parapsilosis*	G1	0.5	<0.015	0.03	0.5	0.5	0.25	WT	ND	FLTLSLRDA	DWIRRYTL
N86	*C. parapsilosis*	G1	0.25	<0.015	0.03	0.25	0.5	0.5	WT	ND	FLTLSLRDA	DWIRRYTL
N87	*C. parapsilosis*	G1	1	0.03	0.25	0.5	1	0.5	C1217A (P406Q)	WT	FLTLSLRDA	DWIRRYTL
N88	*C. parapsilosis*	G1	1	0.06	0.25	1	0.5	1	WT	ND	FLTLSLRDA	DWIRRYTL
N89	*C. parapsilosis*	G1	0.25	<0.015	0.06	0.5	1	1	WT	ND	FLTLSLRDA	DWIRRYTL
N103	*C. parapsilosis*	G1	0.25	0.015	0.06	0.5	0.5	0.25	WT	ND	FLTLSLRDA	DWIRRYTL
N105	*C. parapsilosis*	G1	0.5	<0.015	0.06	0.5	1	0.25	WT	ND	FLTLSLRDA	DWIRRYTL
N106	*C. parapsilosis*	G1	0.5	0.015	0.125	0.5	1	0.5	WT	ND	FLTLSLRDA	DWIRRYTL
N110	*C. parapsilosis*	G1	0.125	<0.015	0.03	0.5	1	0.125	WT	ND	FLTLSLRDA	DWIRRYTL
N114	*C. orthopsilosis*	G1Orth	0.25	0.06	0.03	0.5	2	0.06	WT	ND	FLTLSLRDA	DWVRRYTL
N117	*C. parapsilosis*	G1	0.25	0.015	0.03	2	1	0.125	WT	ND	FLTLSLRDA	DWIRRYTL
N119	*C. parapsilosis*	G1	2	0.06	0.125	1	2	0.5	WT	WT	FLTLSLRDA	DWIRRYTL
N120	*C. parapsilosis*	G1	0.25	<0.015	0.25	0.5	1	0.125	C168T	ND	FLTLSLRDA	DWIRRYTL
N124	*C. parapsilosis*	G1	0.5	0.06	0.125	1	0.5	0.25	G327C (L109F)	ND	FLTLSLRDA	DWIRRYTL
N133	*C. parapsilosis*	G1	0.5	<0.015	0.03	0.5	0.5	0.25	WT	ND	FLTLSLRDA	DWIRRYTL
N134	*C. parapsilosis*	G1	0.25	<0.015	0.03	1	1	0.5	WT	ND	FLTLSLRDA	DWIRRYTL
N135	*C. parapsilosis*	G1	0.06	<0.015	0.5	1	4	0.03	C168T	ND	FLTLSLRDA	DWIRRYTL
N137	*C. parapsilosis*	G1	0.25	<0.015	0.03	0.5	1	0.5	C168T	ND	FLTLSLRDA	DWIRRYTL
N138	*C. parapsilosis*	G1	0.125	<0.015	0.03	0.5	1	1	WT	ND	FLTLSLRDA	DWIRRYTL
N139	*C. parapsilosis*	G1	0.125	<0.015	0.06	0.5	1	2	WT	WT	FLTLSLRDA	DWIRRYTL
N148	*C. parapsilosis*	G1	0.25	0.015	0.06	0.5	1	0.5	G1193T (R398I)	ND	FLTLSLRDA	DWIRRYTL
N158	*C. parapsilosis*	G3	0.25	<0.015	0.03	0.25	0.25	0.5	C168T	ND	FLTLSLRDA	DWIRRYTL
N166	*C. parapsilosis*	G1	0.25	<0.015	0.25	2	2	0.25	WT	ND	FLTLSLRDA	DWIRRYTL
N174	*C. parapsilosis*	G1	0.25	<0.015	0.03	0.5	0.5	0.5	C168T	ND	FLTLSLRDA	DWIRRYTL
N175	*C. parapsilosis*	G1	0.25	<0.015	0.03	0.5	1	0.25	C168T	ND	FLTLSLRDA	DWIRRYTL
N176	*C. parapsilosis*	G2	0.25	<0.015	0.25	0.5	2	0.5	G1193T (R398I)	ND	FLTLSLRDA	DWIRRYTL
N180	*C. parapsilosis*	G1	0.5	0.015	0.03	0.5	2	0.25	WT	ND	FLTLSLRDA	DWIRRYTL
N183	*C. parapsilosis*	G2	0.25	0.015	0.125	1	1	0.125	G1193T (R398I)	ND	FLTLSLRDA	DWIRRYTL
N184	*C. parapsilosis*	G2	0.25	0.015	0.06	1	2	0.125	G1193T (R398I)	ND	FLTLSLRDA	DWIRRYTL
N185	*C. parapsilosis*	G2	0.5	0.015	0.06	1	1	0.125	WT	ND	FLTLSLRDA	DWIRRYTL
N187	*C. parapsilosis*	G1	0.25	<0.015	0.03	0.5	1	0.25	WT	ND	FLTLSLRDA	DWIRRYTL
N193	*C. parapsilosis*	G2	0.25	0.03	0.03	0.5	2	0.125	G1193T (R398I)	ND	FLTLSLRDA	DWIRRYTL
N207	*C. parapsilosis*	G3	0.25	<0.015	0.03	0.5	1	0.5	WT	ND	FLTLSLRDA	DWIRRYTL
N209	*C. parapsilosis*	G3	0.06	<0.015	0.25	1	0.25	0.25	C168T	ND	FLTLSLRDA	DWIRRYTL
N212	*C. parapsilosis*	G1	0.125	<0.015	0.06	0.5	1	0.25	C168T	ND	FLTLSLRDA	DWIRRYTL
N213	*C. parapsilosis*	G1	0.125	<0.015	0.125	0.5	1	0.06	C168T	ND	FLTLSLRDA	DWIRRYTL
N214	*C. parapsilosis*	G1	0.25	0.015	0.06	1	1	0.25	WT	ND	FLTLSLRDA	DWIRRYTL
N215	*C. parapsilosis*	G2	0.25	<0.015	0.03	0.5	1	0.125	G327C (L109F)	ND	FLTLSLRDA	DWIRRYTL
N217	*C. parapsilosis*	G1	0.25	<0.015	0.03	0.5	1	1	C168T	ND	FLTLSLRDA	DWIRRYTL
N220	*C. parapsilosis*	G1	0.25	<0.015	0.03	0.5	1	0.5	WT	ND	FLTLSLRDA	DWIRRYTL
N221	*C. parapsilosis*	G1	0.25	<0.015	0.03	0.5	1	1	C168T	ND	FLTLSLRDA	DWIRRYTL
N222	*C. parapsilosis*	G1	0.5	0.015	0.125	0.5	1	1	WT	ND	FLTLSLRDA	DWIRRYTL
N223	*C. parapsilosis*	G1	0.5	0.015	0.125	1	1	1	C168T	ND	FLTLSLRDA	DWIRRYTL
N225	*C. parapsilosis*	G1	0.125	0.015	0.25	1	0.5	0.25	WT	ND	FLTLSLRDA	DWIRRYTL
N226	*C. parapsilosis*	G3	0.5	<0.015	0.03	1	1	0.25	WT	ND	FLTLSLRDA	DWIRRYTL
33AZ	*C. parapsilosis*	G3	0.5	0.015	0.25	0.5	1	2	G1193T (R398I)	3306-3307 Insertion of T	FLTLSLRDA	DWIRRYTL

* *Located in the first interval of MRR1 (3057–3094), which does not code amino acids*.

*All of the ERG11 sequences harbored the silent mutation of T591C*.

*NA, Not assigned; ND, Not determined; WT, Wild-type; G, Genotype; FLZ, Fluconazole; VRZ, Voriconazole; ITZ, Itraconazole; MFG, Micafungin; AFG, Anidulafungin; and AMB, Amphotericin B*.

### Antifungal Susceptibility Testing

Overall, antifungal resistance was rare. One isolate was multidrug resistant as it was resistant against both FLZ (≥8 μg/ml) and MFG (8 μg/ml) (isolate# 131-BC) (Tables 1, 2). Moreover, intermediate-anidulafungin (*n* = 5, one was the MDR isolate) and –VRZ (*n* = 2) were noted and one isolate was NWT for ITZ (2 μg/ml). ANF showed the highest geometric mean value (1.12 μg/ml), followed by MFG (0.7 μg/ml), FLZ and AMB (each 0.3 μg/ml), ITZ (0.08 μg/ml), and VRZ (0.02 μg/ml) (Table 2).

**Table 2:** Antifungal susceptibility data derived from *C. parapsilosis* species complex isolates in this study.

**Antifungal drugs**	**MIC Values**													**Range**	**GM**	**MIC 50**	**MIC 90**
	**≤0.015**	**0.03**	**0.06**	**0.125**	**0.25**	**0.5**	**1**	**2**	**4**	**8**	**16**	**32**	**≥64**				
FLZ		1	2	9	49	27	7	3			1			0.0625–16 μg/ml	0.3425	0.25	1
VRZ	75	12	7	2	3									<0.015–0.5 μg/ml	0.0154	0.0156	0.0625
ITZ	1	30	19	25	17	6		1						0.0156–2 μg/ml	0.0903	0.125	0.25
MCF					5	56	25	12		1				0.25–8 μg/ml	0.7095	0.5	2
ANF					1	15	53	25	5					0.25–4 μg/ml	1.1263	1	2
AMB		1	5	10	32	27	22	2						0.0312–2 μg/ml	0.3674	0.5	1

*GM, Geometric mean value; FLZ, fluconazole; VRZ, Voriconazole; ITZ, Itraconazole; MCF, Micafungin; ANF, Anidulafungin; AMB, Amphotericin B; MIC, Minimum inhibitory concentration*.

### Sequencing of *ERG11, MRR1*, and *FKS1* HS1 and HS2

None of isolates harbored any silent or nonsynonymous mutations in the *FKS1* HS1 and HS2 (Table 1). However, in *ERG11* the silent mutations T591C, C168T, and G747C were detected in 94, 17, and one isolate(s), respectively (Table 1). As for non-synonymous mutations in *ERG11*, G1193T (R398I) (*n* = 9; 9.5%), A740R (D247G) (*n* = 4; 4.2%), G327C (L109F) (*n* = 2; 2.1%) were the most frequently encountered mutations, followed by G266T (G89V) and C1217A (P406Q) each occurred in one isolate (Table 1 and Supplementary Table 4). None of these mutations were encountered in FLZ-R (≥8 μg/ml) and ITZ-R isolates (>0.5 μg/ml) (Table 1 and Supplementary Tables 4, 5). As Y132F was not found in FLZ-R *ERG11*, the Cp*MRR1* was sequenced for this isolate (131BC) and 11 randomly selected FLZ-S isolates from all three cities (Table 1 and Supplementary Table 5). The MDR isolate did no harbor any mutations in *MRR1*, while for FLZ-S isolates a silent mutation (A231G) occurred in two isolates (isolate# 60-1BC and 60-2BC) and A3080R occurred for one isolate (N1W). Moreover, insertion of a T nucleotide in the position of 3306-3307 was detected in one isolate (isolate# 33AZ) leading to translation termination (Table 1 and Supplementary Table 5).

### Genotyping Diversity Evaluation Using AFLP

**Figure 1:**
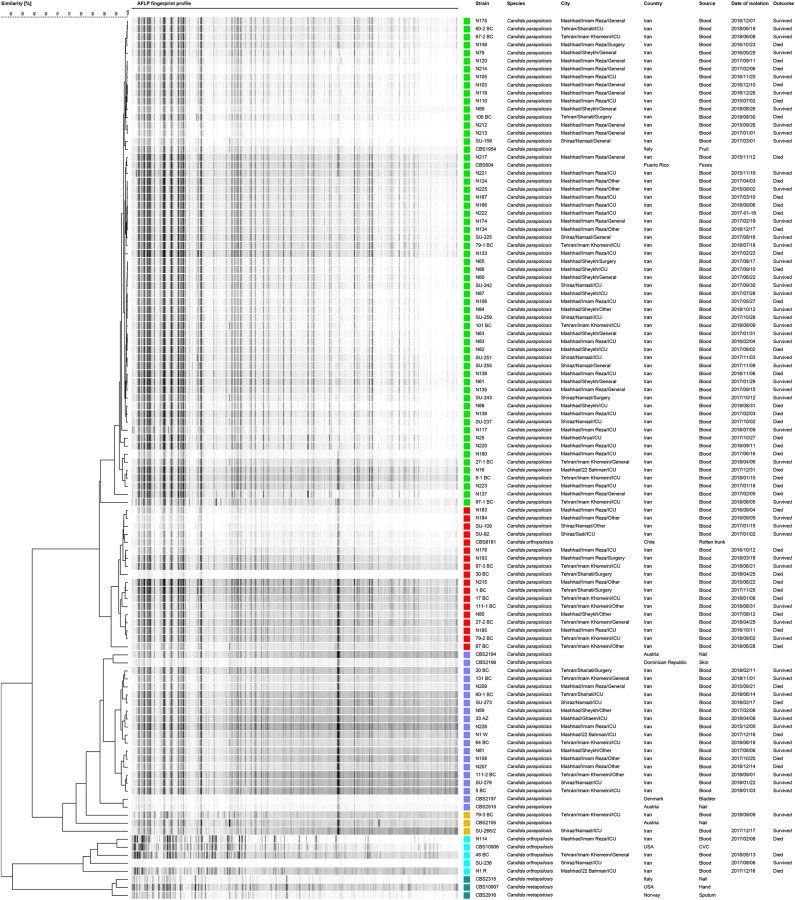
*Candida parapsilosis* isolates showed five distinct genotypes when subjected to AFLP typing, including G1 (green), G2 (red), and G3 (dark blue), and G4 and G5 (Orange). *Candida orthopsilosis* isolates (the reference strain and clinical isolates) and *Candida metapsilosis* reference strains are shown in light blue and dark green colors, respectively. Reference strains *C. parapsilosis, C. orthopsilosis*, and *C. metapsilosis* denoted with CBS numbers were included in AFLP analysis.

AFLP analysis clustered the *C. parapsilosis* isolates (*n* = 94) into five genotypes (G) (Figure 1 and Table 1). G1 was the most abundant genotype (*n* = 59, 62.7%), followed by G2 (*n* = 17, 18%), G3 (*n* = 16, 17%), G4 (*n* = 1, 1.06%), and G5 (*n* = 1, 1.06%) (Figure 1 and Table 1). The vast majority of isolates recovered from Mashhad (*n* = 43, 72.8%) and Shiraz (*n* = 8, 61.5%) grouped in G1, while G2 accommodated almost 35% of isolates (*n* = 8) from Tehran (Figure 1 and Table 1). Except for 97-1BC and 97-2BC clustered in the same genotype, the rest of repetitive isolates were scattered over two (97-3 BC, 27-BC, 111-BC) or even three genotypes (79-BC) (Figure 1 and Table 1). All *C. orthopsilosis* isolates (*n* = 4) showed a distinct genotype, except for one isolate that did not show a decent visible fragment pattern (SU236) (Figure 1, Table 1, Supplementary Table 2). Chi-square analysis did not show any significant association between genotypes and clinical outcome (*P* > 0.2; Supplementary Table 2). Moreover, Kruskal-Wallis Test analysis did not exhibit a significant association between hospitalization duration and genotypes (*P* > 0.489; Supplementary Table 2).

## Discussion

The prevalence of *Candida parapsilosis* in this retrospective study (2015–2019) showed a city-dependent pattern. In Mashhad the prevalence of *C. parapsilosis* (36%) was even higher than that of *C. albicans* (32%) (data not shown and part of ongoing regional epidemiologic studies), while in Tehran and Shiraz this species accounted for the third most common cause of candidemia. Although an Iranian meta-analysis study speculated that *C. parapsilosis* is the leading agent of candidemia (Vaezi et al., 2017), another prospective single-center study from Tehran, Iran, showed that this species accounted for the second agent of candidemia in children (Charsizadeh et al., 2018a). Lack of sufficient number of candidemia studies in Iran prevents the establishment of a clear understanding of candidemia epidemiology separately for children and adults on a national scale. Similar to other studies (McCarty and Pappas, 2016), utilization of broad-spectrum antibiotics, CVC insertion, surgeries (especially abdominal surgeries), mechanical ventilation, and parenteral nutrition were the most important risk factors. Unaffordability of echinocandins in developing countries (Chakrabarti et al., 2015; Singh et al., 2018; Arastehfar et al., 2019b) was the main reason for the extensive use of FLZ in this study, which deviates from the international guidelines that recommend utilization of echinocandins for treatment of candidemia in adults (Hope et al., 2012; Pappas et al., 2016) and AMB (Hope et al., 2012; Pappas et al., 2016) and/or echinocandins for children (Pappas et al., 2016). The lack of adherence to international guidelines is illustrated by the huge observed variation of administered antifungal drug and the dosages used, and the fact that almost 52.2% of cases were left untreated with systemic antifungal drugs (Hope et al., 2012; Pappas et al., 2016). The observed high mortality rate of 46.6% reported in this study is close to values reported in Brazil (Brito et al., 2006; Colombo et al., 2006), the USA (Gudlaugsson et al., 2003), Portugal (Costa-de-Oliveira et al., 2008), and Italy (Tumbarello et al., 2007) with a mortality range of 30–46%, which are in contrast with observations from Taiwan (Wu et al., 2017) and Maryland (Sofair et al., 2006) with a mortality rate of 14%. The surprisingly high death rate observed for those treated with systemic antifungals relative to those not treated (53.2 vs. 36.2%) could be multifactorial and the retrospective nature of our study and the scarcity of detailed clinical data did not allow us to draw a specific conclusion in this regard and prospective, detailed case-control studies are required to clarify this matter.

Adaptation of *C. parapsilosis* to harsh environments (Dogen et al., 2017), being ubiquitously found in man-made and natural environments (Dogen et al., 2017), and the fact this yeast species is predominantly isolated from the hands of HCWs (Delfino et al., 2014) emphasizes the importance of genotyping techniques to find the source of infections and to confine its clonal spread. In this study, AFLP fingerprinting revealed that G1 accounted for almost 60% of candidemia cases. We did not find any significant association between genotypes and mortality and hospitalization duration, whilst mortality was significantly associated with city (*P* 0.02) as Mashhad with the highest mortality rate (56.8%) had the highest number of isolates that showed the least genetic diversity as inferred from AFLP data. The clonal expansion of G1 in Iran in general, and in Mashhad in particular, might be explained by (a) lack of efficient strict hygiene and infection control strategies (Singh et al., 2019), (b) strain-level variation in adhesion and biofilm production abilities leading to survival of a specific tenacious genotype (Silva-Dias et al., 2015), and (c) the lack of true mating type loci in *C. parapsilosis* (Toth et al., 2019). The fact that *C. parapsilosis* was the first cause of candidemia in Mashhad, further indicates the presence of an ongoing outbreak in this city (data not shown and derived from regional epidemiology studies). Of note, obtaining high level of genetic similarity between isolates included in this study, especially those clustered in G1, might be an indication for the lower discriminatory power of AFLP when compared to other typing techniques, such as microsatellite and whole genome sequencing. The genotypic variation observed for serial isolates might have resulted from chromosomal changes, which is accompanied by the emergence of drug resistance in other NAC species, such as *C. glabrata* (Muller et al., 2009; Polakova et al., 2009; Healey et al., 2016). Alternatively, this phenomenon may lead to a better adaptation to the host environment and a higher virulence abilities (Carrete et al., 2019). Additionally, in some cases a given patient might be infected with strains belonging to different genotypes. *C. orthopsilosis* isolates were recovered from all centers involved (*n* = 4) and varied in AFLP fingerprint profiles and showed a higher degree of heterogeneity that could be due to the hybrid nature of this species (Pryszcz et al., 2014). Positivity of one blood samples for both *C. parapsilosis* and *C. orthopsilosis* was extensively discussed in our previous study (Arastehfar et al., 2019a) and confirmed earlier findings (Barbedo et al., 2015) that almost 9.5% of blood isolates contained both species.

Overall, antifungal resistance was rare, but interestingly, we showed that one isolate was MDR (131-BC), which was simultaneously resistant to both FLZ (16 μg/ml) and MFG (8 μg/ml). This phenomenon is paralleled with the recent clonal emergence of MDR *C. parapsilosis* in a pediatric surgery ward in Ege University Hospital, Turkey, in which the MDR isolates were also resistant against both fluconazole and micafungin and harbored Y132F+K143R in Erg11p and R658G in HS1-Fks1p (Arastehfar et al., 2020a). However, we did not find any mutation in *ERG11, MRR1*, and HS1/2 of *FKS1* in our MDR isolate, but we found five non-synonymous mutations in the *ERG11* of FLZ-susceptible isolates, four of them were new (G89V, L109F, D247G, and P406Q) and one (R398I) was previously described from Kuwait (Asadzadeh et al., 2017a) and Korea (Choi et al., 2018). They were not associated with triazole resistance as those mutations were far away from the active site of the enzyme and heme-binding region (Sagatova et al., 2018). Lack of identifying mutations in the genes studied might be explained by the fact that there might be other FLZ and echinocandin-resistant mechanisms involved in addition to what discovered. Indeed at least for azoles, it has been shown that the upregulation of *MDR1, CDR1*, and *ERG11* in *C. parapsilosis* are not merely controlled by gain of function (GOF) mutations in their regulating proteins, i.e., *MRR1, TAC1*, and *UPC2*, respectively (Toth et al., 2019), which further shows the complexity of regulatory networks governing azole resistance in this species.

Surprisingly, the fluconazole and caspofungin therapeutic failures were reported in the Turkish study and the MDR-infected patients were not exposed to echinocandins, but the MDR *C. parapsilosis* isolate harbored R658G in their HS1-Fks1 (Arastehfar et al., 2020a). Therefore, we assessed the previous exposure with echinocandins and azoles and potential antifungal therapeutic failure in our MDR-infected patient. Therapeutic failure was defined if the patient showed persistent fever despite antifungal therapy. Our patient (19-year-old female) was neutropenic, suffered from leukemia, and had a central venous catheter. FLZ therapeutic failure occurred while the patient was on prophylactic FLZ treatment (400 mg PO/day, for 10 days), but survived following CVC removal and caspofungin therapy (loading 70 mg stat/day and then 50 mg stat/day for 5 days).

The overall low rate of antifungal resistance is similar to what is observed in several European countries (Austria, Italy, and Spain) and Asia Pacific countries (Bassetti et al., 2013; Tortorano et al., 2013; Tan et al., 2016; Beyer et al., 2019), but quite different from studies conducted in the USA, South Africa, and India with surprisingly high rates of azole resistance (Raghuram et al., 2012; Govender et al., 2016; Singh et al., 2019). Alarmingly, in the candidemia studies conducted in South Africa (Govender et al., 2016) and Turkey (Hilmioglu-Polat et al., 2018) almost half of FLZ-R isolates were cross-resistant to VRZ. Similar to South Africa (Govender et al., 2016), Turkey (Hilmioglu-Polat et al., 2018), Qatar (Taj-Aldeen et al., 2018), Asia-Pacific countries (Tan et al., 2016), Spain and Italy (Bassetti et al., 2013; Tortorano et al., 2013), and Austria (Beyer et al., 2019) that showed low levels of echinocandin resistance (except for one MCF-R isolate), our isolates were susceptible to this antifungal class. We assume that such a low level of antifungal resistance in our study might be explained by the lack of previous and prolonged antifungal exposure (Ii et al., 2013; Perlin, 2015) and the fact that prophylactic antifungal therapy is not well-exercised in many Iranian hospitals. Surprisingly, as discussed earlier (Arastehfar et al., 2019b), some patients infected with azole/echinocandin susceptible *C. parapsilosis* and *C. orthopsilosis* isolates died despite the use of antifungal therapy, which is in agreement with other studies showing that in both species *in-vitro* susceptibility does not always correlate with clinical outcome (Choi et al., 2010; Wessel et al., 2013; Dimopoulou et al., 2014; Oliveira et al., 2014). This could be viewed as a multifaceted controversial concept that might arise from the underlying condition of the patients, the potent sequestration of azole and echinocandins by biofilm (Soldini et al., 2018), colonization in tissues inhibiting efficient drug penetration (Zhao et al., 2017), and the synergistic antifungal activity of the immune system (Dimopoulou et al., 2014).

We admit that this study could have benefited from the assessment of biofilm production as a mortality predictor and assessment of the expression profiles of *CDR1, ERG11*, and *MDR1*. Although, the application of AFLP fingerprinting in different studies showed species-dependent variations in resolution (Tavanti et al., 2010; Prakash et al., 2016; Asadzadeh et al., 2017b; Restrepo et al., 2018), AFLP cannot differentiate homozygous and heterozygous alleles and may have a lower discriminatory power relative to microsatellite typing and whole genome sequencing (WGS). Therefore, comparing AFLP with other resolutive techniques is of paramount importance to identify the most economic and resolutive typing techniques to be used in clinic.

## Conclusion

Herein, for the first time we reported the molecular epidemiology, antifungal susceptibility testing, and clinical outcomes of Iranian patients suffering from *C. parapsilosis* candidemia and interestingly we found one MDR *C. parapsilosis*. AFLP revealed a high degree of genetic similarity, at least in Mashhad as *C. parapsilosis* was the first cause of candidemia in this city, which may reinforce the importance of application of proper and effective infection control strategies. Moreover, huge variability observed for antifungal drug type and dosages used and the fact that more than half of the patients did not receive any systemic antifungal drugs revealed deviation and lack of compliance with international guidelines.

## Data Availability Statement

The datasets generated for this study can be found in the form of tables, figures and supplemental data. GenBank data obtained for sequencing of genes of interest are included in this study.

## Ethics Statement

The candidemic patients from whom the *C. parapsilosis* isolates were obtained, were recruited to regional candidemia studies conducted in Shiraz, Tehran, and Mashhad. Those studies were approved by ethical committees of Shiraz University of Medical Science (IR.SUMS.REC.1397.365), Tehran University of Medical Sciences (IR. TUMS.SPH.REC.1396.4195), and Mashhad University of Medical Sciences (IR.MUMS fm REC.1397.268). Consent forms were obtained from patients and isolates of *C. parapsilosis* were assigned with numerical codes to anonymize the patient's identity. Written informed consent to participate in this study was provided by the participants' legal guardian/next of kin.

## Author Contributions

AA, SK, KZ, FD, MK, WP, CL-F, and TB designed the study. MN, HZ, KZ, SK, SM, and ZZ provided the clinical isolates. MN, HZ, KZ, and SK obtained ethical approval. MS, SK, KZ, MN, HZ, ZZ, ZN, SK, and SM provided the clinical data. ZN performed the antifungal susceptibility testing. AA and FD designed the primers and performed the sequencing. AA, FD, and FH performed the AFLP. AA, MS, FD, and FH performed the data analysis. AA prepared the first draft. WP, TB, and KZ funded this study. All authors contributed to draft revision.

## Conflict of Interest

MK is an employee of Bruker Daltonik GmbH, Bremen, Germany, the manufacturer of the MALDI-TOF MS system used for Candida identification in the current study. The remaining authors declare that the research was conducted in the absence of any commercial or financial relationships that could be construed as a potential conflict of interest.
