# Supplementary material for: Evaluation of Molecular Epidemiology, Clinical Characteristics, Antifungal Susceptibility Profiles, and Molecular Mechanisms of Antifungal Resistance of Iranian Candida parapsilosis Species Complex Blood Isolates
Source: Front Cell Infect Microbiol. 2020 May 21;10:206. doi: 10.3389/fcimb.2020.00206 (PMC7253641; doi:10.3389/fcimb.2020.00206)
Supplement: Supplementary file 1 [file Data_Sheet_1.docx]

Supplementary Table 1. List of primers used for PCR amplification and sequencing of target genes.

| **Oligo Name** | **Sequence** | **Target gene/Purpose** | **PCR product sizes** | **PCR program** | **PCR ingredients** | **Reference** |
| --- | --- | --- | --- | --- | --- | --- |
| Cp**^A^**-ERG11-Fexternal | AACAAAGATCATACGACTG | *CpERG11*/PCR and sequencing | 1805bps |  |  | This study |
| Cp-ERG11-R1 | TAGTCAAATGTTGATAGG | *CpERG11*/sequencing | --- |  |  | This study |
| Cp-ERG11-F1 | AAGGGTCATGAATTTGTG | *CpERG11*/sequencing | --- |  | 5 μl of 10× PCR buffer (NH_4_^+^, without MgCl2), 2mM of MgCl2, 10 pmol of target primers (Fexternal and Rexternal primers), 0.2 mM of mixed dNTP (dNTP mix, 100mM), and 2.5 units of *Taq* polymerase | This study |
| Cp-ERG11-R2 | GAGGTAATGGCAAGTGTG | *CpERG11*/sequencing | --- | 5min 95^º^C,35 cycles of [ 30sec, 95^º^C; 30sec, 52 ^º^C; and 2min,72^º^C], 8min, 72^º^C |  | This study |
| Cp-ERG11-F2 | AGGAGAAGCAATGAGGAA | *CpERG11*/sequencing | --- |  |  | This study |
| Cp-ERG11-R3 | TTGTATGAGCATAACCTG | *CpERG11*/sequencing | --- |  |  | This study |
| Cp-ERG11-F3 | CCATTACATTCCATATTCA | *CpERG11*/sequencing | --- |  |  | This study |
| Cp-ERG11-Rexternal | CATTCTGCATTAAACCCC | *CpERG11*/PCR and sequencing | 1805bps |  |  | This study |
| Com**^B^**-ERG11-Fexternal | ATGGCATTAGTTGACTTRG | *ComERG11*/PCR and sequencing | 1559bps |  |  | This study |
| Com-ERG1-R1 | AGATGGTGATTTCTGGTT | *ComERG11/*sequencing | --- |  | 5 μl of 10× PCR buffer (NH_4_^+^, without MgCl2), 2mM of MgCl2, 10 pmol of target primers (Fexternal and Rexternal primers for *ERG11* and HS1F/R and HS2F/R), 0.2 mM of mixed dNTP (dNTP mix, 100mM), and 2.5 units of *Taq* polymerase | This study |
| Com-ERG11-F1 | GATATGTTCCGTTGATTA | *ComERG11/*sequencing | --- |  |  | This study |
| Com-ERG11-F2 | AAGATGACTGACCAAGA | *ComERG11/*sequencing | --- | 5min 95^º^C,35 cycles of [ 30sec, 95^º^C; 30sec, 52 ^º^C; and 2min,72^º^C], 8min, 72^º^C |  | This study |
| Com-ERG11-R2 | AGTAGCACTGGTATGTT | *ComERG11/*sequencing | --- |  |  | This study |
| Com-ERG11-Rexternal | CATGTKKCTCTCTTTTCC | *ComERG11*/PCR and sequencing | 1559bps |  |  | This study |
| Co-MRR1-Fexternal | CTGTATGGAGAGTGAGAT | *CPMRR1*/PCR and sequencing | 3850bps |  |  | This study |
| Cp-MRR1-F1 | AAACTGTGTAAAGGCTA | *CPMRR1*/sequencing | --- |  |  | This study |
| Cp-MRR1-F2 | AATTTATCAAACACGAGA | *CPMRR1*/sequencing | --- |  |  | This study |
| Cp-MRR1-F3 | AGAAGAGTTTATCGAGTG | *CPMRR1*/sequencing | --- |  |  | This study |
| Cp-MRR1-F4 | TTTATCAGCGTTGGTTG | *CPMRR1*/sequencing | --- |  |  | This study |
| Cp-MRR1-F5 | TTGACGTATTTTTACTTG | *CPMRR1*/sequencing | --- | 5min 95^º^C,35 cycles of [ 30sec, 95^º^C; 30sec, 52 ^º^C; and 4min,72^º^C], 8min, 72^º^C | 5 μl of 10× PCR buffer (NH_4_^+^, without MgCl2), 2mM of MgCl2, 10 pmol of target primers (Fexternal and Rexternal primers), 0.2 mM of mixed dNTP (dNTP mix, 100mM), and 2.5 units of *Taq* polymerase | This study |
| Cp-MRR1-F6 | GGTCAATTTGTGTGAAG | *CPMRR1*/sequencing | --- |  |  | This study |
| Cp-MRR1-Rexternal | GTTACAGGTGTATAGTGG | *CPMRR1*/PCR and sequencing | 3850bps |  | 5 μl of 10× PCR buffer (NH_4_^+^, without MgCl2), 2mM of MgCl2, 10 pmol of target primers (Fexternal and Rexternal primers), 0.2 mM of mixed dNTP (dNTP mix, 100mM), and 2.5 units of *Taq* polymerase | This study |
| Cp-MRR1-R1 | GAATTTCTGTCTCTGATT | *CPMRR1*/sequencing | --- |  |  | This study |
| Cp-MRR1-R2 | ATTTGTCCTTGAATCAG | *CPMRR1*/sequencing | --- |  |  | This study |
| Cp-MRR1-R3 | CAAGTCTAGTCTTTTCTC | *CPMRR1*/sequencing | --- |  |  | This study |
| Cp-MRR1-R4 | TTCTTTCTCTTATCTGTT | *CPMRR1*/sequencing | --- |  |  | This study |
| Cp-MRR1-R5 | GATAAACATATCACAAATCA | *CPMRR1*/sequencing | --- |  |  | This study |
| Cp-MRR1-R6 | TATCCTTTGATGTCGATG | *CPMRR1*/sequencing | --- |  |  | This study |
| FKS1-HS1-F | CATACRTTTACTGCAAACTTTGT | Cp*FKS1*/PCR and sequencing | 417bps |  |  | This study |
| FKS1-HS1-R | GATTTCCATTTCGGTGGT | Cp*FKS1*/PCR and sequencing | 417bps | 5min 95^º^C,35 cycles of [ 30sec, 95^º^C; 30sec, 52 ^º^C; and 30sec,72^º^C], 8min, 72^º^C | 5 μl of 10× PCR buffer (NH_4_^+^, without MgCl2), 2mM of MgCl2, 10 pmol of target primers (HS1F/R and HS2F/R), 0.2 mM of mixed dNTP (dNTP mix, 100mM), and 2.5 units of *Taq* polymerase | This study |
| FKS1-HS2-F | TGCATRTGAACGAAGATATTTA | Cp*FKS1*/PCR and sequencing | 568bps |  |  | This study |
| FKS1-HS2-R | GCAACAAARACTTCAAACAT | Cp*FKS1*/PCR and sequencing | 568bps |  |  | This study |

1. Those primers specifically were used for *C. parapsilosis*
2. Those primers specifically were used for *C. orthopsilosis*

**Supplementary Table 2 (Statistical analysis)**

**Association of outcome and genotype**

**Association of outcome and G1**

| **Chi-Square Tests** | | | | | |
| --- | --- | --- | --- | --- | --- |
|  | Value | df | Asymptotic Significance (2-sided) | Exact Sig. (2-sided) | Exact Sig. (1-sided) |
| Pearson Chi-Square | .041^a^ | 1 | .840 |  |  |
| Continuity Correction^b^ | .000 | 1 | 1.000 |  |  |
| Likelihood Ratio | .041 | 1 | .840 |  |  |
| Fisher's Exact Test |  |  |  | 1.000 | .507 |
| Linear-by-Linear Association | .040 | 1 | .841 |  |  |
| N of Valid Cases | 88 |  |  |  |  |
| a. 0 cells (0.0%) have expected count less than 5. The minimum expected count is 14.55. | | | | | |
| b. Computed only for a 2x2 table | | | | | |

**Association of outcome and G2**

| **Chi-Square Tests** | | | | | |
| --- | --- | --- | --- | --- | --- |
|  | Value | df | Asymptotic Significance (2-sided) | Exact Sig. (2-sided) | Exact Sig. (1-sided) |
| Pearson Chi-Square | .931^a^ | 1 | .335 |  |  |
| Continuity Correction^b^ | .489 | 1 | .484 |  |  |
| Likelihood Ratio | .928 | 1 | .335 |  |  |
| Fisher's Exact Test |  |  |  | .428 | .242 |
| Linear-by-Linear Association | .921 | 1 | .337 |  |  |
| N of Valid Cases | 88 |  |  |  |  |
| a. 0 cells (0.0%) have expected count less than 5. The minimum expected count is 8.18. | | | | | |
| b. Computed only for a 2x2 table | | | | | |

**Association of outcome and G3**

| **Chi-Square Tests** | | | | | |
| --- | --- | --- | --- | --- | --- |
|  | Value | df | Asymptotic Significance (2-sided) | Exact Sig. (2-sided) | Exact Sig. (1-sided) |
| Pearson Chi-Square | .637^a^ | 1 | .425 |  |  |
| Continuity Correction^b^ | .256 | 1 | .613 |  |  |
| Likelihood Ratio | .647 | 1 | .421 |  |  |
| Fisher's Exact Test |  |  |  | .561 | .309 |
| Linear-by-Linear Association | .630 | 1 | .427 |  |  |
| N of Valid Cases | 88 |  |  |  |  |
| a. 0 cells (0.0%) have expected count less than 5. The minimum expected count is 6.36. | | | | | |
| b. Computed only for a 2x2 table | | | | | |

| **Association of city and outcome Cross- tabulation** | | | | |
| --- | --- | --- | --- | --- |
| Count | | | | |
|  | | outcome | | Total |
|  |  | death | survival |  |
| city | mashhad | 32 | 26 | 58 |
|  | tehran | 6 | 10 | 16 |
|  | shiraz | 2 | 11 | 13 |
| Total | | 40 | 47 | 87 |

| **Chi-Square Tests** | | | |
| --- | --- | --- | --- |
|  | Value | df | Asymptotic Significance (2-sided) |
| Pearson Chi-Square | 7.336^a^ | 2 | .026 |
| Likelihood Ratio | 7.928 | 2 | .019 |
| Linear-by-Linear Association | 7.229 | 1 | .007 |
| N of Valid Cases | 87 |  |  |
| a. 0 cells (0.0%) have expected count less than 5. The minimum expected count is 5.98. | | | |

| **Symmetric Measures** | | | |
| --- | --- | --- | --- |
|  | | Value | Approximate Significance |
| Nominal by Nominal | Phi | .290 | .026 |
|  | Cramer's V | .290 | .026 |
| N of Valid Cases | | 87 |  |

**Logistic Regression analysis was used to assess the association between genotypes and death incidence. Following analysis, none of those genotypes were significantly associated with death.**

| **Variables in the Equation** | | | | | | | |
| --- | --- | --- | --- | --- | --- | --- | --- |
|  | | B | S.E. | Wald | df | Sig. | Exp(B) |
| Step 1^a^ | G1 | -.255 | .630 | .164 | 1 | .686 | .775 |
|  | G2 | -.693 | .742 | .874 | 1 | .350 | .500 |
|  | G3 | .470 | .570 | .680 | 1 | .410 | 1.600 |
| a. Variable(s) entered on step 1: G1, G2,G3 | | | | | | | |

**Association of outcome and Mashhad**

| **Chi-Square Tests** | | | | | |
| --- | --- | --- | --- | --- | --- |
|  | Value | df | Asymptotic Significance (2-sided) | Exact Sig. (2-sided) | Exact Sig. (1-sided) |
| Pearson Chi-Square | 5.923^a^ | 1 | .015 |  |  |
| Continuity Correction^b^ | 4.865 | 1 | .027 |  |  |
| Likelihood Ratio | 6.098 | 1 | .014 |  |  |
| Fisher's Exact Test |  |  |  | .022 | .013 |
| Linear-by-Linear Association | 5.855 | 1 | .016 |  |  |
| N of Valid Cases | 87 |  |  |  |  |
| a. 0 cells (0.0%) have expected count less than 5. The minimum expected count is 13.33. | | | | | |
| b. Computed only for a 2x2 table | | | | | |

**Association of outcome and Tehran**

| **Chi-Square Tests** | | | | | |
| --- | --- | --- | --- | --- | --- |
|  | Value | df | Asymptotic Significance (2-sided) | Exact Sig. (2-sided) | Exact Sig. (1-sided) |
| Pearson Chi-Square | .567^a^ | 1 | .451 |  |  |
| Continuity Correction^b^ | .226 | 1 | .634 |  |  |
| Likelihood Ratio | .574 | 1 | .449 |  |  |
| Fisher's Exact Test |  |  |  | .581 | .319 |
| Linear-by-Linear Association | .561 | 1 | .454 |  |  |
| N of Valid Cases | 87 |  |  |  |  |
| a. 0 cells (0.0%) have expected count less than 5. The minimum expected count is 7.36. | | | | | |
| b. Computed only for a 2x2 table | | | | | |

**Association of outcome and Shiraz**

| **Chi-Square Tests** | | | | | |
| --- | --- | --- | --- | --- | --- |
|  | Value | df | Asymptotic Significance (2-sided) | Exact Sig. (2-sided) | Exact Sig. (1-sided) |
| Pearson Chi-Square | 5.759^a^ | 1 | .016 |  |  |
| Continuity Correction^b^ | 4.402 | 1 | .036 |  |  |
| Likelihood Ratio | 6.350 | 1 | .012 |  |  |
| Fisher's Exact Test |  |  |  | .018 | .015 |
| Linear-by-Linear Association | 5.693 | 1 | .017 |  |  |
| N of Valid Cases | 87 |  |  |  |  |
| a. 0 cells (0.0%) have expected count less than 5. The minimum expected count is 5.98. b. Computed only for a 2x2 table | | | | | |

**Association of genotypes and hospitalization duration Cross-tabulation were tested by Chi-Square Test. Multiple isolates with various genotyepes were excluded from measurements to prevent bias. As the hospitalization duration values were not normally distributed, therefore, the association of hospitalization duration and genotypes were calculated using Kruskal-Wallis Test.**

| **One-Sample Kolmogorov-Smirnov Test** | | |
| --- | --- | --- |
|  | | Hospitalizaton duration |
| N | | 83 |
| Normal Parameters^a,b^ | Mean | 47.08 |
|  | Std. Deviation | 59.263 |
| Most Extreme Differences | Absolute | .218 |
|  | Positive | .175 |
|  | Negative | -.218 |
| Test Statistic | | .218 |
| Asymp. Sig. (2-tailed) | | .000^c^ |
| a. Test distribution is Normal. | | |
| b. Calculated from data. | | |
| c. Lilliefors Significance Correction. | | |

| **Test Statistics^a,b^** | |
| --- | --- |
|  | Hospitalizaton duration |
| Chi-Square | 1.431 |
| df | 2 |
| Asymp. Sig. | .489 |
| a. Kruskal Wallis Test | |
| b. Grouping Variable: Genotype | |

Percentage and numbers used for statistical analysis.

| Supplementary Table 3. The summary of clinical data from patients presented in this study | | | |
| --- | --- | --- | --- |
| Variable |  | N | % |
| Total number of: | Isolates | 98 |  |
|  | Patients | 90 |  |
| Total number of patients with candidemia due to | *Candida parapsilosis* | 86 | 95.6 |
|  | *Candida orthopsilosis* | 3 | 3.3 |
|  | *C. parapsilosis* and *C. orthopsilosis* (simultaneously) | 1 | 11.1 |
| Sex | Female | 45 | 50 |
|  | Male | 45 | 50 |
| Age | Median | 34.5 yr |  |
|  | Children | 35 | 38.9 |
|  | Adults | 55 | 61.1 |
| City | Tehran | 17 | 20.7 |
|  | Shiraz | 14 | 15.2 |
|  | Mashhad | 59 | 64.1 |
| Hospital ward | ICU | 44 | 48.9 |
|  | General | 24 | 26.7 |
|  | Surgery | 8 | 8.9 |
|  | Other | 14 | 15.6 |
| Hospitalization duration | Median | 39 d |  |
|  | ≤30 | 40 | 44.4 |
|  | 30-90 | 39 | 43.3 |
|  | >90 | 11 | 12.2 |
| Risk factors | | | |
| CVC | Yes | 78 | 86.7 |
|  | No | 12 | 13.3 |
| Surgery | Abdominal surgery | 14 | 15.6 |
|  | Non-abdominal surgery | 16 | 17.8 |
|  | Without surgery | 60 | 66.7 |
| Parental nutrition | Yes | 25 | 27.8 |
|  | No | 65 | 72.2 |
| Mechanical ventilation | Yes | 35 | 38.9 |
|  | No | 55 | 61.1 |
| Immunosuppressive drugs | Yes | 12 | 13.3 |
|  | No | 78 | 86.7 |
| Neutropenia | Yes | 15 | 16.7 |
|  | No | 75 | 83.3 |
| Underlying conditions | | | |
| Leukemia | Yes | 11 | 12.2 |
|  | No | 79 | 87.8 |
| Hematological malignancies other than leukemia | Yes | 2 | 2.2 |
|  | No | 88 | 97.8 |
| Diabetes | Yes | 19 | 21.1 |
|  | No | 71 | 78.9 |
| Dialysis | Yes | 6 | 6.7 |
|  | No | 84 | 93.3 |
| Sepsis | Yes | 10 | 11.1 |
|  | No | 80 | 88.9 |
| Cerebral events | Yes | 5 | 5.6 |
|  | No | 85 | 94.4 |
| Burning | Yes | 6 | 6.7 |
|  | No | 84 | 93.3 |
| Vascular and heart events | Yes | 16 | 17.8 |
|  | No | 74 | 82.2 |
| Chronic lung diseases | Yes | 15 | 16.7 |
|  | No | 75 | 83.3 |
| Solid tumor | Yes | 8 | 8.9 |
|  | No | 82 | 91.1 |
| Trauma and fracture | Yes | 2 | 2.2 |
|  | No | 88 | 97.8 |
| Abdominal events | Yes | 19 | 21.1 |
|  | No | 71 | 78.9 |
| Viral infections | Yes | 5 | 5.6 |
|  | No | 85 | 94.4 |
| Other | Yes | 13 | 14.4 |
|  | No | 77 | 85.6 |
| Treatment and mortality | | | |
| Overall mortality rate  (Total N=90) | Died | 42 | 46.7 |
|  | Survived | 48 | 53.3 |
| Mortality rate for *C. parapsilosis*  (N=87), considering the mixed infection | Died | 40 | 46 |
|  | Survived | 47 | 54 |
| Mortality rate for *C. parapsilosis* per city (N=87) | Tehran | 6/16 | 37.5 |
|  | Shiraz | 2/13 | 15.4 |
|  | Mashhad | 32/58 | 54.2 |
| Mortality rate for *C. parapsilosis*  (N=86), without mixed infection | Died | 39 | 45.3 |
|  | Survived | 47 | 54.7 |
| Mortality rate for *C. parapsilosis* per city (N=86) | Tehran | 6/16 | 37.5 |
|  | Shiraz | 2/13 | 15.4 |
|  | Mashhad | 31/57 | 54.4 |
| Mortality rate for *C. orthopsilosis*  (N=3) | Died | 2 | 66.7 |
|  | Survived | 1 | 33.3 |
| Mortality rate for *C. orthopsilosis* per city (N=3) | Tehran | 1/1 | 100 |
|  | Shiraz | 0/1 | 0 |
|  | Mashhad | 1/1 | 100 |
| Mortality for mixed infection  (N=1) | Died | 1/1 | 100 |
|  | Survived | 0/1 | 0 |
| Mortality per city | Tehran | 7/17 | 41.2 |
|  | Shiraz | 2/14 | 14.3 |
|  | Mashhad | 33/59 | 55.9 |
| Mortality per duration | ≤30 | 18/40 | 45 |
|  | 30-90 | 21/39 | 53.8 |
|  | >90 | 3/11 | 27.3 |
| Treatment  (monotherapy versus combination therapy) | Monotherapy | 31 | 34.4 |
|  | Treated with >1 antifungal | 12 | 13.3 |
|  | No or topical treatment | 47 | 52.2 |
| Treatment  (systemic treatment versus no or topical treatment) | Systemic antifungal therapy (including monotherapy and combination therapy) | 43 | 47.8 |
|  | No or topical antifungal treatment | 47 | 52.2 |
| Treatment (when used as monotherapy)  Total=31 | FLZ | 15 | 48.4 |
|  | AMB | 9 | 29 |
|  | CAS | 6 | 19.3 |
|  | VRZ | 1 | 3.2 |
| Mortality per treatment  (monotherapy versus those treated with >1 antifungal) | Monotherapy | 17/31 | 54.8 |
|  | Combination therapy | 8/12 | 66.7 |
|  | No or topical treatment | 17/47 | 36.2 |
| Mortality per treatment  (systemic treatment versus no or topical treatment) | Systemic antifungal therapy (including monotherapy and combination therapy) | 25/43 | 58.1 |
|  | No or topical treatment | 17/47 | 36.2 |
| Mortality per treatment (when drugs used as monotherapy) | FLZ | 10/15 | 66.7 |
|  | AMB | 6/9 | 66.7 |
|  | CAS | 1/6 | 16.7 |
|  | VRZ | 0/1 | 0 |
| Abbreviations: FLZ: fluconazole, AMB: amphotericin B, CAS: caspofungin, VOR: voriconazole, CLT: clotrimazole, NSY: nystatin | | | |

Supplementary Table 4.Frequency of encountered non-synonymous mutations in *ERG11* along with the wild-type and the fluconazole MIC values for corresponding *C. parapsilosis* isolates.

| **Fluconazole MIC values (µg/ml)** | | | | | | | | | |  |  |  |  |
| --- | --- | --- | --- | --- | --- | --- | --- | --- | --- | --- | --- | --- | --- |
| ***ERG11* Mutation** | **S%** | **R%** | **≤0.125** | **0.25** | **0.5** | **1** | **2** | **4** | **8** | **16** | **32** | **64** | **Total #** |
| Wild-type | 98.8% | 1.2% | 10 | 36 | 23 | 6 | 3 |  |  | 1 |  |  | 79 |
| G266T (G89V) | 100% | 0.00% |  |  | 1 |  |  |  |  |  |  |  | 1 |
| G327C (L109F) | 100% | 0.00% |  | 1 | 1 |  |  |  |  |  |  |  | 2 |
| A740R (D247G) | 100% | 0.00% |  | 3 | 1 |  |  |  |  |  |  |  | 4 |
| G1193T (R398I) | 100% | 0.00% |  | 8 |  |  |  |  |  |  |  |  | 8 |
| C1217A (P406Q) | 100% | 0.00% |  |  |  | 1 |  |  |  |  |  |  | 1 |

Supplementary Table 5. Frequency of *C. parapsilosis* isolates along with mutations in *ERG11* and *MRR1* and the MIC values in corresponding strains carrying those mutations.

| ***ERG11*** | ***MRR1*** | **Voriconazole MIC values (µg/ml)** | | | | | |  | **Itraconazole MIC values (µg/ml)** | | | | | |
| --- | --- | --- | --- | --- | --- | --- | --- | --- | --- | --- | --- | --- | --- | --- |
|  |  | ≥0.015 | 0.03 | 0.06 | 0.125 | 0.25 | 0.5 |  | ≥0.015 | 0.03 | 0.06 | 0.125 | 0.25 | 0.5 |
| G266T (G89V) |  | 1 |  |  |  |  |  |  |  | 1 |  |  |  |  |
| G327C (L109F) |  | 1 |  | 1 |  |  |  |  |  | 1 |  | 1 |  |  |
| A740R (D247G) |  | 4 |  |  |  |  |  |  |  | 1 | 1 |  | 1 | 1 |
| G1193T (R398I) |  | 6 | 1 |  |  |  |  |  |  | 2 | 2 | 2 | 1 |  |
| G1193T (R398I) | 3306-3307 Insertion of T | 1 |  |  |  |  |  |  |  |  |  |  | 1 |  |
| C1217A (P406Q) |  |  | 1 |  |  |  |  |  |  |  |  |  | 1 |  |
|  | A3080R (Q1027R) |  | 1 |  |  |  |  |  |  |  |  |  |  | 1 |
